# More Kindness, Less Prejudice against Immigrants? A Preliminary Study with Adolescents

**DOI:** 10.3390/ejihpe13010017

**Published:** 2023-01-16

**Authors:** Ioana Zagrean, Lucrezia Cavagnis, Francesca Danioni, Claudia Russo, Maria Cinque, Daniela Barni

**Affiliations:** 1 Department of Human Sciences, LUMSA University of Rome, 00193 Rome, Italy; 2 Department of Human and Social Sciences, University of Bergamo, 24129 Bergamo, Italy; 3 Family Studies and Research University Centre, Catholic University of Milan, 20123 Milan, Italy

**Keywords:** prejudice against immigrants, kindness, adolescents’ sex

## Abstract

Prejudice against immigrants is a relevant research topic within social psychology. Researchers identified several individual variables affecting anti-immigrant prejudice, such as morality and personality. However, until now, prejudice has never been studied in relation to kindness, which might be a significant protective factor against prejudice. Based on Kohlberg’s theory of moral judgement, four stage dimensions of kindness were identified, from egocentric to authentic kindness (i.e., a means for social progress and improvement). This study aims to explore the relationship between the four kindness dimensions and blatant and subtle prejudice against immigrants in adolescence, by also considering the moderating role of adolescents’ sex. It involved 215 Italian participants (77% girls), who were asked to fill in a self-report questionnaire. Results showed that boys scored higher on egocentric kindness than girls, but no sex differences emerged for prejudice. Egocentric and extrinsically motivated kindness appeared to be risk factors for prejudice, whereas the most authentic form of kindness was a protective factor. In addition, adolescents’ sex moderated the relationship between egocentric kindness and blatant prejudice, whereby this association was stronger for boys. The implications of these findings, the study’s limitations, and suggestions for future research are discussed.

## 1. Introduction

Ethnic prejudice is a relevant research topic within social psychology because of its many consequences on individual quality of life, interpersonal relationships, and social functioning. The first conceptualization was made by Allport [1], who defined prejudice as “an antipathy based upon a faulty and inflexible generalization” (p. 9). Ethnic prejudice specifically refers to a set of negative beliefs, attitudes, and judgments about whole categories of people because of their different ethnic backgrounds and about individual members of these categories.

In some ways, ethnic prejudice was more evident in the past compared to nowadays. Suffice it to say that racial laws were promulgated before and during World War II and enforced racial discrimination toward the Jewish population; moreover, legal slavery in the USA was abolished only in 1865. Specific discrimination laws based on the ethnic background of people were preserved for a long time (e.g., black people were not allowed to sit in the section of the bus reserved for white people) [2].

Evidence concerning the change of prejudice over time is somehow contrasting. On the one hand, it has been suggested that ethnic prejudice has decreased. For example, Myers [3] noted that in 1942 only approximately one-third of a pool of white respondents agreed with the idea of having mixed classes in schools (with both white and black students), whereas in 1980, the agreement raised to about 90%. On the other hand, there is evidence that prejudice is still a current issue. For example, research showed that the former president of the US, Barack Obama, obtained 6% fewer votes than expected because of prejudice [4]. Also, when reflecting on real-life episodes of ethnic prejudice, George Floyd’s case immediately comes to our mind, together with the documented evidence suggesting that black people are more likely to get shot by police officers in ambiguous situations (see the so-called “shooter bias”) [5]. Interestingly, most discriminated people are aware of the discrimination itself, also in neutral daily life situations, such as buying goods at the market [6]. Research confirms that this perception is usually correct: people who perceive discrimination are actually victims of discrimination [7]. Eibach and Ehrlinger explained the contrasting results concerning increased vs. decreased levels of ethnic prejudice over time by showing that white people tend to compare today’s levels of prejudice with the past, whereas black people tend to compare the present time to their ideal world [8]. Indeed, ethnic prejudice keeps being a social problem as it can lead to discrimination and hate crimes [9,10], such as intimidation, property damage, or even assault or murder.

More recent research has suggested that the expression of prejudice has changed over time. Indeed, scholars suggest that there are other forms of ethnic prejudice in addition to the more overt form. Consistently, Pettigrew and Mertens proposed the well-known distinction between “blatant prejudice” and “subtle prejudice” [11]. Blatant prejudice concerns open rejection, beliefs of genetic inferiority, and highly discriminatory behaviours. Instead, subtle prejudice deals with the defence of traditional values, exaggeration of cultural differences, and denial of positive emotions towards people coming from other countries. Concisely, they described blatant prejudice as “hot, close and direct”, while subtle prejudice was described as “cool, distant and indirect” [11] (p. 58).

Adolescence is a crucial stage for the development of ethnic prejudice [12,13], so it is paramount to study prejudice during this developmental stage. Several individual factors (e.g., personality, ideologies, and social dominance orientation) and contextual factors (e.g., family, friends, and school) play a significant role in shaping adolescents’ prejudice. In this specific life phase, substantial attention has been paid to socialization contexts. In line with Bandura’s [14] social learning theory, parents can act as modelling agents and contribute to shaping adolescents’ views [15]. Thus, negative attitudes against immigrants and ethnic minorities can be intergenerationally transmitted [16,17].

Similarly, the role of peers is crucial for understanding adolescents’ prejudice. Indeed, friends are usually similar in their attitudes towards immigrants and ethnic minorities, and they serve as role models for peers [15]. Furthermore, different theoretical frameworks (i.e., intergroup contact theory and educational theories) underlined that multiple factors related to the school experience could influence adolescents’ prejudice. Thijs and Verkuyten [18] highlighted that the school environment plays a relevant role in experiencing interethnic contact, and Wölfer et al. [19] found that cross-ethnic friendship can reduce prejudice. Additionally, those schools that promote inclusion and cultural pluralism contribute to significantly reducing students’ prejudice [20].

Despite the research showing that substantial attention has been paid to socialization and contextual factors in the development of adolescents’ prejudice, individual factors are also important. For example, Miglietta and colleagues [21] showed that social dominance orientation contributes to increase the levels of adolescents’ ethnic prejudice (see also [22]). Moreover, Duriez [23] showed that adolescents’ right-wing authoritarianism was positively associated with ethnic prejudice. Additionally, Korol [24] found that multicultural personality dimensions, such as empathy and open-mindedness, were positively associated with ethnic tolerance in adolescence. Ethnic tolerance consists of “a set of attitudes toward equality of people who are culturally or racially different” [24] (p. 265) and, to some extent, it can be interpreted as the counterpart of ethnic prejudice.

Ethnic prejudice also appeared to be significantly related to morality [25]. In their recent work, Hoover and colleagues [26] found that the extreme behavioural expressions of prejudice (EBEPs) (e.g., hate speech and hate group activity) partially depend on moral values. They suggest that EBEPs can originate from people’s values and their perception of moral violations. Basically, the ingroup’s moral values are associated with the outgroup’s EBEPs: when the ingroup believes that the outgroup has done something that is morally wrong, this is likely to affect prejudice towards the outgroup, thus leading to EBEPs.

Among others, kindness is highly connected to morality, but it has never been studied in relation to ethnic prejudice. According to Collins’ English dictionary, kindness is the quality of being gentle, caring, and helpful. Within the scientific literature, there is a substantial disagreement about the definition of kindness and a paucity of works on the topic. Canter and colleagues [27] suggest that kindness is a multifaceted interpersonal trait composed of three dimensions (i.e., benign tolerance, empathetic responsivity, and principled proaction), which converge in a general kindness dimension (i.e., core kindness). Exline and colleagues [28] consider it as a set of social norms concerning how people should behave within society, whereas Lyubomirsky and colleagues [29] define kindness as a behaviour that benefits others even at the expense of the self.

As stated before, kindness is related to morality and is considered a moral virtue [30]. In this regard, Comunian [31] studied kindness by adopting a specific theoretical framework based on both the development of morality and the cognitive developmental approach [32,33,34]. According to this framework, values and ethics develop as subsequent stages in a continuous exchange between individuals and their environment. Accordingly, kindness is not seen as a continuum (from completely unkind to extremely kind) but as hierarchical developmental stages. In the first stage, kindness can be defined as *egocentric and subjective*. Here, people tend to be focused primarily on their personal interests and goals. People follow the rules just to avoid trouble, and others’ expectations are not considered. In the second stage, people can consider others’ perspectives, but kindness is still based on self-interest; in this stage, kindness is defined as *social/normative*. In the third stage, people consider others’ perspectives and recognize the importance of mutuality and social satisfaction. Here, kindness can be considered as *extrinsically motivated* since a kind behaviour is mainly driven by external factors (e.g., reaching social satisfaction, supporting sense of belonging, etc.). In the last developmental stage, the perspective of others is considered, as well as societal and symbolic ones. Kindness is considered important for positive communication with others, and it is internalized as a value. At this stage, kindness could be defined as *authentic*.

Research has clearly shown that kindness can act as a protective factor in different life domains. It increases subjective happiness and satisfaction with life [29] while also reducing anxiety levels, physical symptoms such as the common cold, and even high blood pressure [35]. Additionally, it promotes positive social interactions [36]. Considering these results and the positive role of kindness in interpersonal relations and its closeness to morality [25,30], the aim of the present study was to explore how the four different stages of kindness presented above are related to prejudice against immigrants. As known, this is a specific form of ethnic prejudice, which is particularly relevant in the increasingly multicultural society where adolescents are growing up. We also explored whether and the extent to which adolescents’ sex plays a moderating role in the relationship between kindness and prejudice against immigrants. There is a paucity of empirical studies on adolescents’ sex differences in kindness and prejudice. However, the available literature on the adult population has shown that women are generally kinder [37,38,39,40] and show less prejudice than men [41,42,43,44].

Building on the above, we hypothesized that egocentric kindness should show a positive relationship with blatant and subtle prejudice against immigrants (H1a). In contrast, authentic kindness should be negatively associated with prejudice (H1b). We did not formulate any specific hypothesis about social/normative and extrinsically motivated kindness, because of the lack of available literature on the topic. Based on the literature involving adults, we could expect that girls show higher levels of kindness (H2), especially of the most evolved form (i.e., authentic kindness), and lower levels of prejudice (H3) than boys. Additionally, we expected that the association between kindness and prejudice against immigrants could be moderated by sex (H4). Once again, we cannot formulate any specific hypothesis about the role of sex, given the total absence of previous studies on the topic.

## 2. Method

### 2.1. Participants and Procedure

Two hundred fifteen high school students (77% girls) aged between 15 and 19 (*M* = 16.7, *SD* = 0.72) took part in this study. Among the participants, 76.3% lived with both their parents, 18% lived only with the mother, and 1.9% with the father; 3.8% reported “other” as a response. Most participants were Italian (97%) and were born and raised in Italy (98%). Data were collected through the collaboration of different high schools attended by students with different backgrounds, mostly located in central Italy (80.8%). Within each school, some classes were randomly selected, and all the students of those classes were invited to a preliminary online meeting where the principal investigator explained the main objectives of the study, the survey procedure, and the participants’ rights. Participants were informed of their rights, including the right to refuse to participate or to withdraw from the study at any time. Adolescents, or their parents if adolescents were underage, provided written informed consent. Neither schools nor families received any incentive for participation.

Students who agreed to participate in the study and whose parents gave their consent were asked to fill in an anonymous online survey, receiving the link to the questionnaire by e-mail. We ensured that all the participants had a computer, tablet, or smartphone and an internet connection. In some cases, the school provided a tablet or similar connection device to those students who did not have it. In addition, we controlled for careless responding bias by improving respondents’ participation engagement to reduce the risk of nonresponses and checking for random answers. Following the guidelines of Ward and Meade [45], to limit this bias, we added to the survey some instructions that made salient the amount of work and time needed to develop a questionnaire. On the same webpage, participants were asked to fill out three statements related to their commitment to the survey (i.e., I acknowledge that this study will take approximately 20/25 minutes; I am aware that my participation will enable the advancement of scientific knowledge; I undertake to read each question carefully and answer truthfully). We also added a control item in the middle of the survey (i.e., ‘This is a control item. Please select “absolutely agree”’) [45].

The entire procedure was in accordance with the Declaration of Helsinki [46] and with the ethical guidelines for research provided by the Italian Psychological Association [47]. The study was approved by the Ethics Committee of the LUMSA University (protocol nr. 7/2021).

### 2.2. Measures

#### 2.2.1. Sociodemographic Information

Respondents were asked to provide sociodemographic data (sex, age, country of birth, nationality) and information about family characteristics (cohabiting family members).

#### 2.2.2. Kindness

We used the Kindness Scale [31] composed of 20 items measuring the four types of kindness based on Kohlberg’s developmental stages of moral judgement [32]. Each subscale includes five items. Stage 1 refers to a type of kindness characterized by egocentrism and subjectivity (item example: “I make others believe that I am listening to them”, α = 0.89), while Stage 2 considers kindness as an ability to see reciprocal relations (item example: “I am kind with people who were good to me”, α = 0.58). At Stage 3, kindness is extrinsically motivated, with people believing that social satisfaction and understanding must be mutual to be effective (item example: “I like to appear as kind as others seem to be”, α = 0.65). Finally, at Stage 4, people base their interactions on a societal perspective-taking (item example: “I am kind because I believe in respecting the dignity of others”, α = 0.77). Participants responded on a 4-point Likert scale (from 1 = not at all true to 4 = completely true). The score of each type of kindness was calculated by averaging the relative items: the higher the score, the higher the level of kindness.

#### 2.2.3. Blatant and Subtle Prejudice

We used the Italian version of the Blatant and Subtle Prejudice Scale (BSPS) [11], which was translated and validated in Italian by Manganelli Rattazzi and Volpato [48]. The scale is a 20-item measure; 10 items measure blatant prejudice (item example: “Immigrants and Italians can never be really comfortable with each other, even if they are close friends”, α = 0.87), and the other 10 measure subtle prejudice (item example: “Values that immigrants teach to their children are different from those taught by Italians”, α = 0.67). Participants responded on a 7-point Likert scale (from 1 = completely disagree to 7 = completely agree). The scores were calculated by averaging the relative items: the higher the score, the higher the level of blatant or subtle prejudice.

### 2.3. Data Analysis

We conducted an a priori sample size calculation in G*Power [49] using the *F*-test (multiple linear regression), an α of 0.05, and a power of 0.95. We set 9 as the number of tested predictors and the total number of predictors (adolescents’ sex, the four types of kindness, and their two-way interactions with sex). The effect size was set as medium (Cohen’s *f*^2^ of 0.15). The analysis provided a required minimum sample size of 167 participants.

In the first step of the analysis, we checked for the normality of variables’ distribution. Specifically, we considered skewness and kurtosis, which should be in the range of −1 to +1 [50]. Second, we reported descriptive statistics (mean, SD, and range) and Pearson correlations among the study variables.

To examine the relationship between kindness and prejudice, and whether this was moderated by participants’ sex, we tested two multiple regression models. The four kindness types were entered as the predictors, sex as the moderator, and blatant or subtle prejudice as the dependent variable. The continuous predictors were grand-mean centered to reduce multicollinearity [51]. We conducted the analyses using the GAMLj [52] package for Jamovi [53].

## 3. Results

The descriptive statistics of the variables are shown in Table 1. To explore sex differences on kindness scores, a Mann–Whitney test was conducted since the number of boys and girls was unbalanced. The only significant sex difference was found for egocentric kindness *U*(213) = 2868, *p* < 0.001, with boys (*M* = 2.06; *SD* = 0.84) scoring higher than girls (*M* = 1.63; *SD* = 0.70). Boys and girls reported similar scores of social/normative, *U*(213) = 4067, *p* = 0.88, extrinsically motivated, *U*(213) = 4065, *p* = 0.88, and authentic kindness, *U*(213) = 3758, *p* = 0.34. Further, boys and girls did not show statistically significant differences either for blatant (*p* = 0.86) or for subtle (*p* = 0.65) prejudice (see the regression results, Table 3).

As shown in Table 2, egocentric and extrinsically motivated kindness positively correlated with blatant and subtle prejudice, whereas authentic kindness negatively correlated with both of them, despite the fact that these correlations were smaller in size. Social/normative kindness showed a small positive correlation only with subtle prejudice.

### 3.1. Blatant Prejudice

The first moderation regression analysis, with blatant prejudice as dependent variable, showed that the model was significant, *F*(9205) = 9.94, *p* < 0.001, and explained about one third of the variance, adjusted R^2^ = 0.273. As reported in Table 3, the main effect of sex was not significant. Among kindness dimensions, the main effects of egocentric and extrinsically motivated kindness were significant and positive (B = 0.72, *p* < 0.001 and B = 0.50, *p* < 0.05, respectively): The higher were these kindness dimensions, the higher was adolescents’ blatant prejudice. On the contrary, the main effect of social/normative kindness was significant but negative (B = −0.37, *p* < 0.05), thus indicating that the higher this type of kindness was, the lower was adolescents’ blatant prejudice. The only statistically significant moderating effect of sex was found with reference to egocentric kindness. 

**Figure 1:**
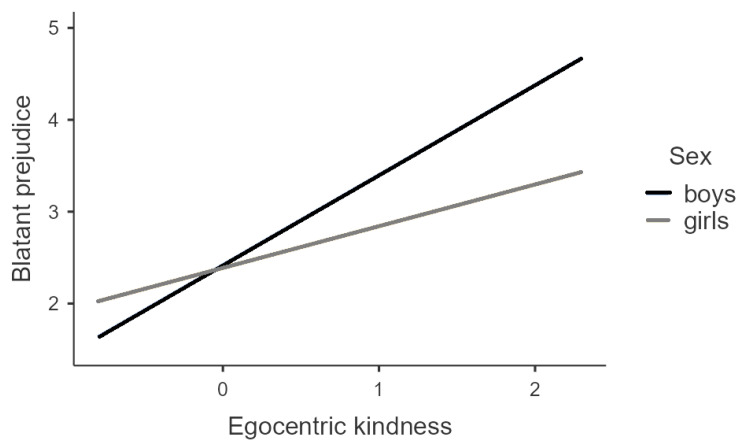
The Moderating Role of Adolescents’ Sex in the Relationship Between Egocentric Kindness and Blatant Prejudice.

The results of simple slopes analysis (Figure 1) pointed out that the association of egocentric kindness with blatant prejudice was stronger for boys (B = 0.98, *p* < 0.001) than for girls (B = 0.45, *p* < 0.001).

### 3.2. Subtle Prejudice

The second moderation analysis, with subtle prejudice as dependent variable, showed that the model was statistically significant, *F*(9205) = 5.96, *p* < 0.001, although this model explained less variance than the previous one (adjusted R^2^ = 0.173). As shown in Table 3, the main effect of sex was not significant; instead, significant effects emerged for kindness. More precisely, egocentric and extrinsically motivated kindness were positively related to subtle prejudice (B = 0.29, *p* < 0.001 and B = 0.47, *p* < 0.005, respectively), while authentic kindness was negatively associated with prejudice (B = −0.54, *p* < 0.001). No statistically significant moderating effects of sex emerged.

## 4. Discussion

Socialization contexts, such as family, school, and friend groups, are highly relevant in the development of prejudice in adolescence [15]. In contrast, less is known about individual factors that could be related to adolescents’ negative attitudes towards immigrants and ethnic minorities. Considering risk and protective factors of prejudice in adolescence is especially relevant because this is a critical period for the formation of general attitudes and the development of prejudicial attitudes.

Kindness was found to play a relevant role in satisfaction with life and in positive social interactions [40,54]. Further, it is also associated with empathy and morality [55,56]. Although prejudice against immigrants is strongly related to morality and moral values [26], to the best of our knowledge this is the first study on the relationship between adolescents’ kindness and prejudice towards immigrants. Additionally, due to the sex differences on kindness and prejudice levels, we explored whether this relationship was moderated by adolescents’ sex.

From the present study, descriptively, it emerged that adolescents showed high scores of social/normative and extrinsically motivated kindness, higher than for the egocentric type. Authentic kindness was at the top of their “hierarchy” of kindness dimensions. One possible interpretation of such results can be made in light of adolescents’ level of moral development, with most of them having reached a social-order-maintaining orientation to foster harmonious relationships among group members. We found that girls showed lower levels of egocentric kindness than boys, but unlike our second hypothesis (H2), no sex differences emerged with regard to authentic kindness. That is, boys are more prone than girls to keep a self-oriented form of kindness, based on the desire for rewards and self-satisfaction. In their study, Kalsoom et al. [57] found that adolescent boys are less care oriented than girls, and, referring to Gilligan’s and Kohlberg’s theories, they suggested that men and women develop differently in morality. Men morally develop through a sense of justice, while women morally develop through caring for others. This other-orientation, stronger for girls [58], is rooted mainly in the cultural environment.

From our results, it emerged that sex was not related to prejudice against immigrants. Contrary to our expectations (H3) and some previous studies [42,43], sex did not affect either blatant or subtle prejudice. However, it is worthwhile to note that not all the previous studies on anti-immigrant prejudice are consistent in reporting sex differences [59] and that the levels of prejudice might be influenced by the target’s sex, which we did not control in our study. In particular, the level of prejudice could differ whether the target of prejudice is of the same sex or of the opposite sex of the person whose prejudice is being measured, as people might show less prejudice toward same-sex others than toward opposite-sex others [44].

The main aim of the present study was to analyze the relationship between kindness and anti-immigrant prejudice. The results showed that kindness could be a risk factor for prejudicial attitudes towards immigrants in adolescence. Furthermore, partially supporting our first hypothesis, we found that egocentric and extrinsically motivated kindness had a significant and positive relationship with both blatant and subtle prejudice (H1a). In other words, adolescents who act kindly for personal interests (i.e., egocentric kindness) or to achieve social satisfaction (i.e., extrinsically motivated kindness) showed higher levels of prejudice.

Social/normative and authentic kindness can instead be a protective factor against specific forms of prejudice. On the one hand, adolescents who behave kindly to be appreciated by others (i.e., social/normative kindness) showed lower levels of blatant prejudice, which is the least socially acceptable form of prejudice. On the other hand, in line with our H1b, authentic kindness is negatively associated with subtle prejudice, whose pervasive and unaware nature makes it more resistant to change. That is, contrasting this inconspicuous, indirect, and often unconscious prejudice needs a genuine kindness resulting from a mature morality. According to Comunian [31], in Stage 4, adolescents recognize kindness as a value and believe in reciprocity. Similarly, a higher level of moral development is associated with lower prejudice [60]. Thus, higher and more sophisticated levels of moral development might drive genuine kindness that, in turn, is associated with lower prejudice against immigrants.

As far as the fourth hypothesis (H4) was concerned, this was substantially disconfirmed. The relationships between kindness and prejudice were similar for boys and girls, with the only exception of the relation between egocentric kindness and blatant prejudice link. Male adolescents showed a stronger positive association between egocentric kindness and blatant prejudice than their female counterparts. This result could be read in terms of personality research. For example, boys generally show stronger Dark Triad traits than girls, such as Machiavellianism [44], which can covary with the level of egocentric kindness, playing a possible confounding role. In essence, it cannot be excluded that high levels of egocentric kindness are associated with high levels of Machiavellianism, and both could lead to increased prejudice. Of course, future research should explore this interpretation more in depth and expand the attention to further possible gendered characteristics.

This study has some limitations, which need to be considered when interpreting the results. First, the study was cross-sectional, meaning that it was not possible to draw causal inferences from the results. Second, our sample was of convenience and was recruited from high schools. However, in the data collection, we involved several schools of different types attended by students with different backgrounds. Third, cross-cultural differences cannot be explored since we only relied on an Italian sample.

## 5. Conclusions

Despite the limitations of the present study, it is the first attempt to analyze the relation between different types of kindness and prejudice towards immigrants among adolescents. This is a particularly crucial topic in the increasingly multicultural society. All in all, the results showed how social/normative and authentic kindness might be considered protective factors against adolescents’ prejudice towards immigrants. Here, others’ perspective is viewed as important, and kindness is internalized and authentic. Interestingly, its role depends on the form of prejudice considered. In particular, authentic kindness, backgrounded in a higher level of morality, can help in protecting against the most challenging form of prejudice, namely, subtle prejudice. 

Differently, egocentric kindness and extrinsically motivated kindness are risk factors for adolescents’ blatant and subtle prejudice towards immigrants. This is especially true for boys, whose egocentric kindness is strongly related to blatant prejudice.

These results confirm that there are different “kindnesses” and clearly suggest that kindness cannot be simply considered a sort of panacea for reducing prejudice. It is not effective in contrasting prejudice to enhance kindness “tout court” among adolescents, but it is necessary to deepen the meaning of kindness. The meaning of kindness must be considered in interventions aimed at contrasting adolescents’ prejudice by strengthening morality, empathy, and social interaction skills. Reciprocity and mutuality seem to be promising qualities of kindness to focus on in trainings aimed at reducing prejudice.

It would be worthwhile for future research to replicate our results in different cultural contexts. Also, longitudinal designs should be employed to investigate the role of kindness in different developmental stages across life. Finally, regarding the influence of sex differences on kindness and prejudice, we definitively need more research on participants with or without immigration background.

## Figures and Tables

**Table 1:** Descriptive Statistics of Kindness and Prejudice.

	Sex	Mean	SD	Range	
Egocentric kindness	Boys	2.06	0.84	1.00	4.00
	Girls	1.63	0.70	1.00	4.00
Social/Normative kindness	Boys	2.96	0.47	1.80	4.00
	Girls	2.94	0.52	1.60	4.00
Extrinsically motivated kindness	Boys	2.93	0.50	1.80	4.00
	Girls	2.95	0.54	1.60	4.00
Authentic kindness	Boys	3.21	0.47	2.20	4.00
	Girls	3.29	0.48	2.00	4.00
Blatant prejudice	Boys	2.74	1.23	1.00	5.20
	Girls	2.34	1.04	1.00	5.20
Subtle prejudice	Boys	3.84	0.80	1.50	5.80
	Girls	3.60	0.78	1.80	5.80

**Table 2:** Pearson Correlations between Kindness and Prejudice.

	1.	2.	3.	4.	5.	6.
1. Egocentric kindness	—					
2. Social/normative kindness	0.28 ***	—				
3. Extrinsically motivated kindness	0.10	0.35 ***	—			
4. Authentic kindness	−0.23 ***	0.27 ***	0.52 ***	—		
5. Blatant prejudice	0.46 ***	0.00	0.18 **	−0.16 *	—	
6. Subtle prejudice	0.37 ***	0.15 *	0.14 *	−0.17 *	0.60 ***	—

*Note*. * *p* < 0.05, ** *p* < 0.01, *** *p* < 0.001.

**Table 3:** Moderation Regression Analyses Results. Outcome variables: Blatant and Subtle Prejudice.

	Blatant Prejudice						Subtle Prejudice					
			95% CI						95% CI			
Variables	B	SE	Lower	Upper	*β*	*p*	B	SE	Lower	Upper	*β*	*p*
Egocentric kindness	0.72	0.10	0.51	0.92	0.50	<0.001	0.29	0.08	0.13	0.45	0.28	<0.001
Social/normative kindness	−0.37	0.19	−0.74	0.00	−0.17	0.048	0.19	0.14	−0.10	0.47	0.12	0.198
Extrinsically motivated kindness	0.50	0.20	0.10	0.89	0.24	0.015	0.47	0.16	0.16	0.78	0.31	0.003
Authentic kindness	−0.32	0.21	−0.73	0.10	−0.14	0.131	−0.54	0.16	−0.86	−0.22	−0.33	<0.001
Sex	−0.03	0.16	−0.35	0.29	−0.03	0.860	−0.06	0.12	−0.30	0.19	−0.07	0.648
Egocentric kindness ∗ Sex	−0.53	0.21	−0.94	−0.12	−0.36	0.012	−0.08	0.16	−0.39	0.24	−0.07	0.637
Social/normative kindness ∗ Sex	0.08	0.38	−0.66	0.83	0.04	0.821	−0.25	0.29	−0.82	0.32	−0.16	0.389
Extrinsically motivated kindness ∗ Sex	0.31	0.40	−0.49	1.10	0.15	0.450	−0.48	0.31	−1.10	0.13	−0.32	0.124
Authentic kindness ∗ Sex	−0.30	0.42	−1.13	0.52	−0.13	0.467	0.42	0.32	−0.21	1.05	0.26	0.193

## Data Availability

The dataset generated during and analyzed during the current study is not publicly available because of local legal and privacy restrictions (Italian Data Protection Code—Legislative Decree No. 196/2003). However, the raw data will be made available upon reasonable request to the corresponding author.
